# Unveiling VEXAS Syndrome: When Skin Manifestations and Monoclonal Gammopathy Precede Myeloid‐Lineage Hematologic Abnormality

**DOI:** 10.1002/acr2.70064

**Published:** 2025-05-30

**Authors:** Laura Di Centa, Simone Longhino, Valeria Manfrè, Stefania Sacco, Luca Quartuccio

**Affiliations:** ^1^ Division of Rheumatology, Department of Medicine University of Udine, Academic Hospital “Santa Maria della Misericordia,” Udine Italy

## Abstract

VEXAS (vacuoles, E1 enzyme, X‐linked, autoinflammatory, somatic) syndrome is a rare disorder caused by somatic *UBA1* gene mutations, characterized by autoinflammation and hematologic abnormalities, particularly affecting myeloid‐lineage progenitors. Sensitive markers include macrocytic anemia, vacuolization of bone marrow precursors, and myelodysplasia. Here, we report the first case of VEXAS syndrome presenting with neutrophilic dermatosis and a serum monoclonal component, without myeloid‐lineage hematologic abnormalities atonset. This case underscores the importance of including VEXAS syndrome in the differential diagnosis when monoclonal gammopathy is associated with rheuamtologic features, particularly in older patients presenting with unexplained cutaneous inflammatory manifestations and a serum clonal component, as such presentations may precede the development of classical hematologic abnormalities.

## Introduction

VEXAS (vacuoles, E1 enzyme, X‐linked, autoinflammatory, somatic) syndrome is a rare adult‐onset disorder caused by somatic loss‐of‐function mutations in the *UBA1* gene in hematopoietic progenitor cells.^1^ These mutations impair the E1 ubiquitin‐activating enzyme, resulting in a heterogeneous clinical spectrum that combines autoinflammatory features, such as fever, neutrophilic dermatosis, chondritis, and serositis, with myeloid‐lineage hematologic abnormalities, including macrocytic anemia, myelodysplastic syndrome (MDS), and bone marrow vacuolization.^2^ Here, we describe a case of VEXAS syndrome initially presenting with autoinflammatory symptoms and a new detected serum monoclonal component in the absence of classic myeloid‐lineage hematologic abnormalities. The authors affirm that human participants provided informed consent for publication of the images. Written informed consent was obtained from all individual participants included in the study.

## Case report

**Figure 1:**
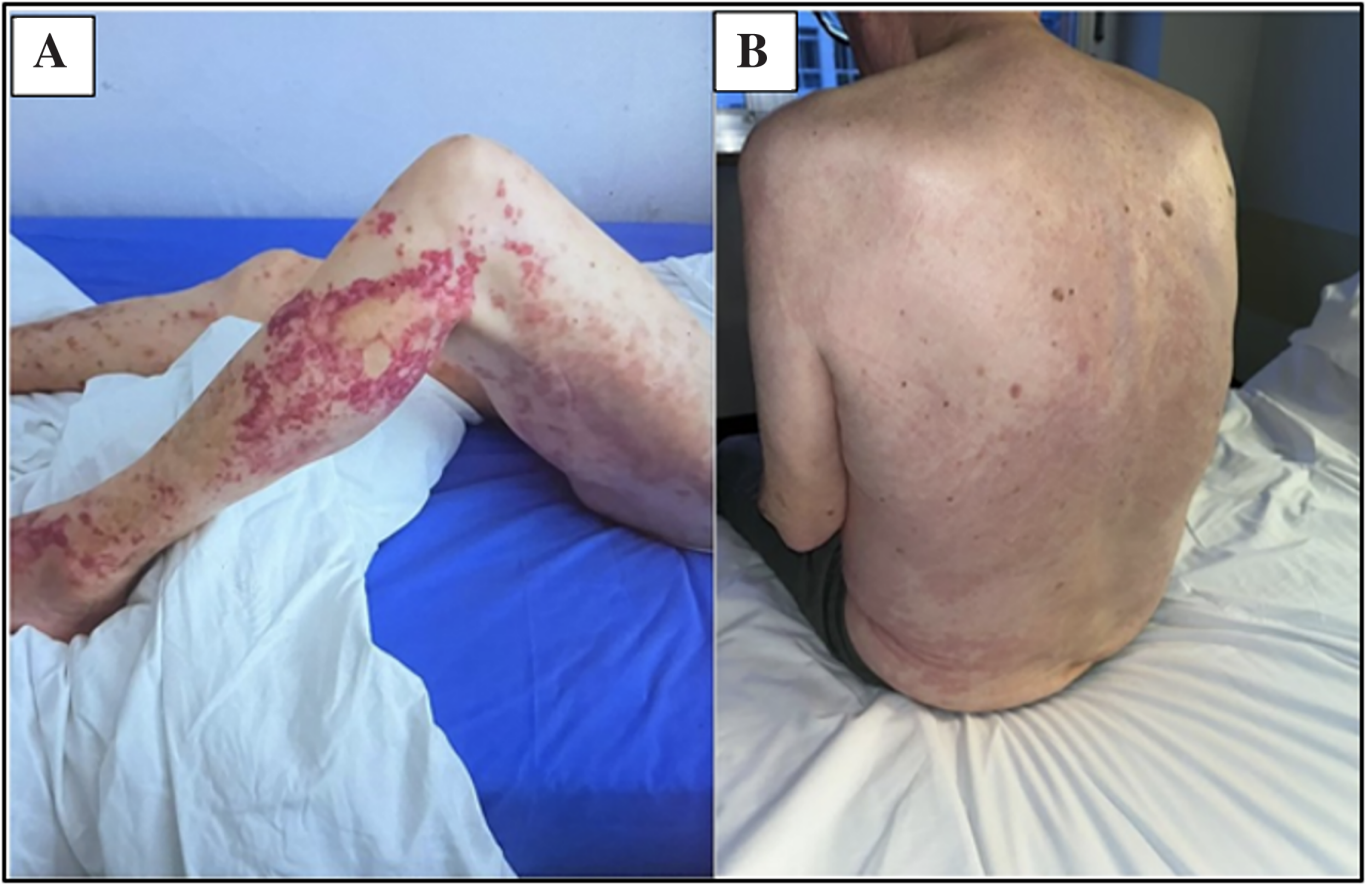
Itchy rash on (A) the lower limbs and on (B) the back as it presented in early stages of disease.

**Figure 2:**
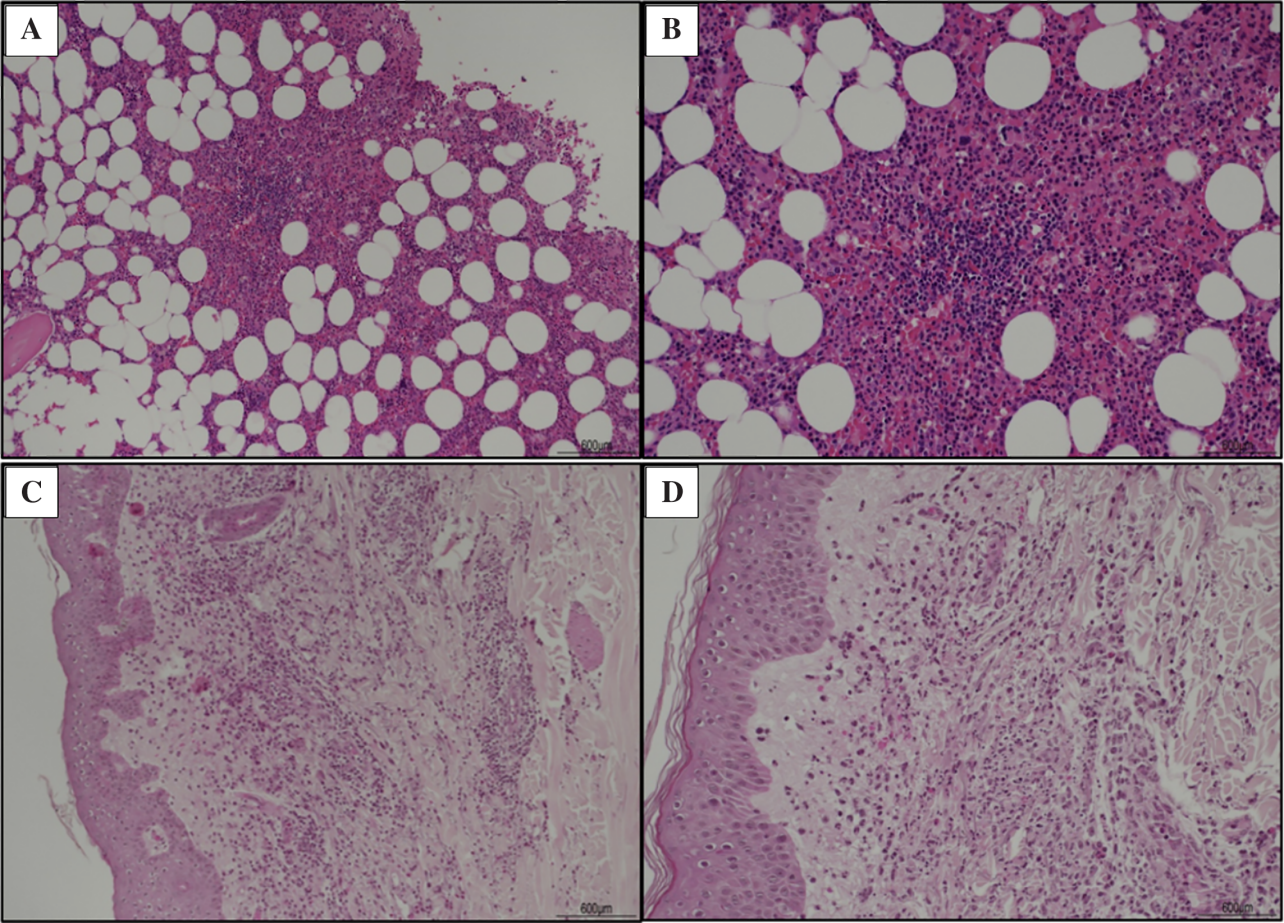
(A and B) Bone marrow biopsy (staining: hematoxylin and eosin) at (A) original magnification ×10 and (B) original magnification ×20. The hematopoietic parenchyma appears hypercellular, with an increase in all hematopoietic lineages, which exhibit normal maturation stages without atypia. Plasma cells are present in normal proportions and show no cytologic abnormalities. No evidence of vacuolization in bone marrow progenitor cells or signs of myelodysplasia is observed. (C and D) Skin biopsy of the left arm (staining: hematoxylin and eosin) at (C) original magnification ×10 and (D) original magnification ×20. A diffuse, interstitial, and perivascular neutrophilic infiltrate with signs of degranulation is present in the dermis, consistent with neutrophilic dermatosis. No evidence of vasculitis is observed. Additionally, interstitial eosinophils, small perivascular lymphocytes, and papillary dermis edema are noted.

The patient, an 80‐year‐old man with no significant medical history, presented in May 2023 with fever (38.5°C), weight loss, asthenia, and an urticarial rash on the lower limbs and back (Figure 1). Laboratory tests revealed elevated markers of inflammation (C‐reactive protein level 250 mg/L, ferritin level 2,500 ng/mL) and a previously unrecognized mild serum IgG λ clonal component. Complete blood cell count, lactate dehydrogenase, renal and hepatic function, and autoimmune tests were unremarkable. A diagnosis of eczema was made following a skin biopsy, and a short course of steroids was initiated, providing only temporary relief. In August 2023, the patient developed pericarditis, which was successfully treated with colchicine. Whole‐body computed tomography and positron emission tomography scans, performed to exclude an occult malignancy, showed no pathologic findings. Because of the persistence of symptoms and the emergence of a new serum clonal component, a bone marrow biopsy was performed but showed no abnormalities (Figure 2A and B). The patient remained steroid dependent until further deterioration in February 2024 prompted a second skin biopsy, which identified neutrophilic dermatosis (Figure 2C and D). Dermatologists initiated dapsone, without significant improvement. In September 2024, after rheumatologic evaluation at our center, Schnitzler syndrome (SchS) was diagnosed based on chronic urticaria, serum monoclonal IgG, fever, elevated markers of inflammation, and neutrophilic dermal infiltrate on the biopsy specimen. Anakinra at 100 mg daily was started but proved ineffective, with recurrence of fever accompanied by severe injection site reactions. By December 2024, mild macrocytic anemia (hemoglobin level 10.7 g/dL, mean corpuscular volume 101 fL) developed. Because of the failure of anakinra, the emergence of macrocytic anemia, and an atypical IgG monoclonal component for SchS, *UBA1* sequencing was performed, revealing the p.Met41Thr mutation, thus establishing a diagnosis of VEXAS syndrome.

## Differential diagnosis

The patient's clinical picture clearly falls within the spectrum of autoinflammatory disorders, given the concurrent presence of fever, elevated markers of inflammation, and chronic urticaria with histologic evidence of neutrophilic dermatosis.^3^ Considering the advanced age and negative autoimmune test results, the main differential diagnoses included SchS and VEXAS syndrome. Initial suspicion leaned toward SchS because of the following: (1) the presence of a serum clonal component, fulfilling the Strasbourg diagnostic criteria; (2) the absence of macrocytic anemia and MDS, which are usually present at the onset of nearly all VEXAS syndrome cases; and (3) the absence of bone marrow vacuolization.^1^, ^2^, ^3^ However, atypical features, including IgG rather than IgM monoclonal gammopathy and pericarditis, which is uncommon in SchS, challenged the diagnosis.^3^, ^4^ Furthermore, the poor response to anakinra, which is generally effective in SchS, and the development of significant injection site reactions, known to occur in VEXAS syndrome, prompted diagnostic reconsideration.^2^, ^3^ The subsequent emergence of macrocytic anemia, despite the absence of bone marrow vacuolization, led to *UBA1* sequencing, confirming VEXAS syndrome.

## Management and outcomes

Following the confirmation of VEXAS syndrome, the patient initiated canakinumab at 300 mg every four weeks. However, the response was only partial, with recurrent fever necessitating narrowing of the dosing interval to every three weeks.

## Discussion

In recent years, there has been growing interest in the category of monoclonal gammopathies of clinical significance (MGCS) with rheumatologic features.^5^ Although SchS, characterized by aberrant inflammasome activation with excessive interleukin‐1β (IL‐1β) production and typically IgM clonal gammopathy, is a well‐recognized entity within this group, VEXAS syndrome, has not yet been classified as such.^5^ This is largely because, although it can present with autoinflammatory manifestations primarily cutaneous in more than 80% of cases, its defining hallmark lies in myeloid‐lineage hematologic abnormalities.^1^, ^2^ However, this case suggests that VEXAS syndrome may also be considered within the spectrum of MGCS with rheumatologic features. In VEXAS syndrome, a serum clonal component is found in about 20% of cases, typically associated with the p.Met41Val mutation; chondritis; gastrointestinal, cardiac, and pulmonary involvement; and increased risk of MDS.^2^ This case suggests a serum clonal component may also appear early in the disease course, even with isolated cutaneous manifestations and delayed myeloid involvement, and in association with other mutations, such as p.Met41Thr. Recognizing a newly detected serum clonal component as a potential early indicator of VEXAS syndrome, particularly in older adults presenting with autoinflammatory manifestations, is crucial, as timely and accurate diagnosis enables a more appropriate therapeutic approach. Indeed, although both SchS and VEXAS syndrome share autoinflammatory features, SchS generally responds well to IL‐1 inhibitors, whereas VEXAS syndrome may exhibit a less favorable response and even significant adverse reactions, making alternative cytokine‐targeted therapies preferable for its management.^2^, ^3^

## Conclusions

This case highlights VEXAS syndrome as a diagnostic consideration in older patients with unexplained autoinflammation, even in the absence of myeloid abnormalities at onset. Given its association with a serum clonal component, VEXAS syndrome may also fit within the spectrum of monoclonal gammopathies with rheumatologic features. Early *UBA1* mutation testing is essential for timely diagnosis and appropriate management, as lymphoid clonal abnormalities may precede classic hematologic manifestations.

## AUTHOR CONTRIBUTIONS

All authors contributed to at least one of the following manuscript preparation roles: conceptualization AND/OR methodology, software, investigation, formal analysis, data curation, visualization, and validation AND drafting or reviewing/editing the final draft. As corresponding author, Dr Quartuccio confirms that all authors have provided the final approval of the version to be published and takes responsibility for the affirmations regarding article submission (eg, not under consideration by another journal), the integrity of the data presented, and the statements regarding compliance with institutional review board/Declaration of Helsinki requirements.

## Supporting information


**Disclosure form**.
